# Structural Covariance Networks in the Fetal Brain Reveal Altered Neurodevelopment for Specific Subtypes of Congenital Heart Disease

**DOI:** 10.1161/JAHA.124.035880

**Published:** 2024-10-25

**Authors:** Siân Wilson, Daniel Cromb, Alexandra F. Bonthrone, Alena Uus, Anthony Price, Alexia Egloff, Milou P. M. Van Poppel, Johannes K. Steinweg, Kuberan Pushparajah, John Simpson, David F. A. Lloyd, Reza Razavi, Jonathan O'Muircheartaigh, A. David Edwards, Joseph V. Hajnal, Mary Rutherford, Serena J. Counsell

**Affiliations:** ^1^ Research Department of Early Life Imaging, School of Biomedical Engineering and Imaging Sciences King’s College London London United Kingdom; ^2^ Fetal‐Neonatal Neuroimaging & Developmental Science Center Boston Children’s Hospital Boston MA USA; ^3^ Division of Newborn Medicine Boston Children’s Hospital Boston MA USA; ^4^ Department of Pediatrics, Harvard Medical School Boston MA USA; ^5^ Biomedical Engineering Department, School of Biomedical Engineering and Imaging Sciences King’s College London London United Kingdom; ^6^ Department of Congenital Heart Disease Evelina London Children’s Hospital London United Kingdom; ^7^ Centre for Neurodevelopmental Disorders King’s College London London United Kingdom; ^8^ Department of Forensic and Neurodevelopmental Sciences King’s College London London United Kingdom

**Keywords:** brain, congenital heart disease, fetal, magnetic resonance imaging, Magnetic Resonance Imaging (MRI), Congenital Heart Disease

## Abstract

**Background:**

Altered structural brain development has been identified in fetuses with congenital heart disease (CHD), suggesting that the neurodevelopmental impairment observed later in life might originate in utero. There are many interacting factors that may perturb neurodevelopment during the fetal period and manifest as structural brain alterations, such as altered cerebral substrate delivery and aberrant fetal hemodynamics.

**Methods and Results:**

We extracted structural covariance networks from the log Jacobian determinants of 435 in utero T2 weighted image magnetic resonance imaging scans, (n=67 controls, 368 with CHD) acquired during the third trimester. We fit general linear models to test whether age, sex, expected cerebral substrate delivery, and CHD diagnosis were significant predictors of structural covariance. We identified significant effects of age, sex, cerebral substrate delivery, and specific CHD diagnosis across a variety of structural covariance networks, including primary motor and sensory cortices, cerebellar regions, frontal cortex, extra‐axial cerebrospinal fluid, thalamus, brainstem, and insula, consistent with widespread coordinated aberrant maturation of specific brain regions over the third trimester.

**Conclusions:**

Structural covariance networks offer a sensitive, data‐driven approach to explore whole‐brain structural changes without anatomical priors. We used them to stratify a heterogenous patient cohort with CHD, highlighting similarities and differences between diagnoses during fetal neurodevelopment. Although there was a clear effect of abnormal fetal hemodynamics on structural brain maturation, our results suggest that this alone does not explain all the variation in brain development between individuals with CHD.

**Nonstandard Abbreviations and Acronyms**
ICA: independent component analysis
SCN: structural covariance network

**Research Perspective**

**What Is New?**

- This data‐driven analysis framework offers an alternative approach to studying a heterogenous patient group, highlighting brain regions that mature differently in utero in this vulnerable population.
- The significant effect of cerebral substrate delivery and congenital heart disease diagnosis on specific brain morphometry supports the view that a lower oxygen environment can lead to altered brain development.

**What Question Should Be Addressed Next?**

- These data also highlight variation between individuals not attributable to fetal substrate delivery, supporting the hypothesis that in utero brain development in congenital heart disease is affected by a complex combination of factors that need further investigation.

Congenital heart disease (CHD) is the most common congenital malformation occurring at a frequency of 0.8% to 1.2% of live births worldwide.^1^, ^2^ CHD encompasses a wide spectrum of cardiovascular defects, from simple cardiac malformations to more complex and severe lesions that require surgical intervention. Following improvements in surgical and therapeutic intervention over the past 2 decades, most patients with CHD survive into adulthood.^3^ However, many display impairments affecting a wide range of neurodevelopmental domains.^4^, ^5^, ^6^, ^7^, ^8^

Recent work has highlighted that the structural underpinnings of abnormal neurodevelopment are present prenatally in the CHD population. In utero magnetic resonance imaging (MRI) studies have identified reduced regional brain volumes,^9^, ^10^, ^11^, ^12^, ^13^, ^14^ smaller transient fetal compartments such as the subplate and intermediate zones,^15^, ^16^ abnormal cortical folding patterns,^17^, ^18^ and a positive correlation between cerebral oxygen delivery and fetal brain size.^14^, ^19^ It has been hypothesized that aberrant cardiovascular physiology impedes the delivery of oxygen, glucose, and other nutrients to the fetal brain during a period of rapid brain growth, which features metabolically demanding cellular processes such as gyrification, oligodendrocyte maturation, and synaptogenesis.^19^, ^20^ There are a multitude of interacting factors that may perturb neurodevelopment during the fetal period and manifest as structural brain alterations. These include cardiac physiology and closely related cerebrovascular hemodynamics,^19^, ^20^, ^21^ genetic factors,^22^, ^23^ placental function,^24^, ^25^ maternal stress,^26^, ^27^ and socioeconomic and environmental factors.^28^ Disentangling the effect of each of these factors remains a challenge, and therefore our understanding of structural brain abnormalities in the CHD population is incomplete.

In this study, we focus on understanding the effect of “cerebral substrate delivery” on brain development, which refers to the substrate content of blood reaching the fetal brain, including oxygen, glucose, and other nutrients.^29^, ^30^, ^31^ In typically developing fetuses, there is preferential streaming of oxygen and nutrient rich placental blood to the fetal brain. In CHD, different subtypes have varying effects on the streaming patterns in the fetal circulatory system, which in turn affect the delivery of substrate‐rich blood to the developing brain.^15^, ^19^, ^29^ For example, in lesions where there is only 1 functional outlet to the heart, such as hypoplastic left heart syndrome (HLHS), there is mixing of placental and fetal systemic venous blood, reducing the substrate availability of blood reaching the brain.^21^ For fetuses with transposition of the great arteries (TGA), there is a complete reversal of normal fetal streaming patterns, with systemic venous return comprising the majority of cerebral blood flow.^21^

We explore the effect of cerebral substrate delivery and specific CHD diagnoses on fetal structural brain development by using independent component analysis (ICA) to extract structural covariance networks (SCNs) from the Jacobian determinants of the T2‐weighted (T2w) brain MRI. Jacobian determinants represent how the relative volumes of different brain structures vary across a population, in a data‐driven manor without anatomical priors.^32^, ^33^ Jacobian‐derived SCNs represent brain regions with coherent structural expansion and coordinated, independent maturational trajectories. For imaging analysis of the fetal brain, which is characterized by highly dynamic contrast change and the presence of transient brain structures, this approach is advantageous over image segmentation tools, that constrain the analysis to a set of predefined structures.

To the best of our knowledge, this study is the first use of SCNs to study in utero structural brain development, although they have been used previously to investigate changes in the organization of the brain at the network level throughout lifespan, often converging on functional brain networks.^34^, ^35^ Previous applications include both healthy aging populations^34^, ^36^, ^37^ and at‐risk populations such as preterm infants,^38^, ^39^ psychiatric patient cohorts,^40^, ^41^ and in neurodegenerative disease.^35^, ^42^

We extracted SCNs from a large cohort of 429 fetuses, including 368 fetuses with CHD, with 2 main aims: (1) to explore how the morphometry of the fetal brain is affected by altered cerebral substrate delivery and (2) to stratify a heterogenous patient cohort with CHD according to differences in brain development. We hypothesized that with this approach, we would be able to disentangle the impact of specific cardiac defects on the developing fetal brain.

## METHODS

The data that support the findings of this study are available from the corresponding author upon reasonable request.

### Ethical Approval

The National Research Ethics Service West London committee provided ethical approval (07/H0707/105, 14/LO/1806, 17/LO/0292). Informed written consent was obtained before fetal MRI.

### Participants

The cohort of fetuses used in this study have been reported on recently.^14^ Briefly, mothers were recruited between June 2014 and June 2022 at the Evelina Children's Hospital in London, following routine ultrasound during the second trimester. The recruitment of control subjects was done through the intelligent Fetal Imaging and Diagnosis project (https://www.ifindproject.com/). The recruitment of subjects with CHD was done separately, by obtaining informed written consent from mothers who were referred for a clinical cardiac MRI scan. Referrals for a clinical cardiac MRI scan undergo neuroimaging as part of the examination and all participants were asked whether they would be willing for the brain images to be used in research. Eighty‐nine percent of women consented for the fetal brain MRI to be used in research studies (percentage of women who consented as a proportion of total clinical scans following a suspected CHD diagnosis 529 consenting women [maternal age scan=31.52 (±5.73) years]) were carrying a fetus with either known or suspected CHD. Participants were scanned during the third trimester, between 27 and 36 gestational weeks. Exclusion criteria for mothers included multiple pregnancies, maternal weight >125 kg (scanner bed weight limit), inability to give informed consent, or age <18 years at the time of referral. We also excluded fetuses with confirmed genetic diagnosis such as 22q deletion syndrome, extracardiac anomalies such as congenital diaphragmatic hernia or duodenal atresia, or structural brain abnormalities reported on fetal MRI, including bilateral ventriculomegaly, cerebellar hypoplasia, or absence of the corpus callosum. After applying the exclusion criteria, the cohort consisted of 435 fetal MR scans (including 67 controls, and 368 fetuses diagnosed with CHD).

### Image Acquisition and Reconstruction

All scans were acquired on a Philips Ingenia 1.5T scanner, with 28‐channel dStream anterior and posterior built‐in coils. The T2w fast‐spin‐echo sequence was specifically optimized for fetal imaging (repetition time =13 milliseconds, echo time=80 milliseconds, image resolution=1.25×1.25×2.5 mm, slice thickness=2.5 mm, slice spacing=1.25 mm). A 3‐dimensional slice‐to‐volume image reconstruction pipeline (available at https://hub.docker.com/r/fetalsvrtk/svrtk auto2.20) was used for motion correction, reconstructing the T2w images to 0.5 mm^3^ isotropic resolution.^43^, ^44^

### MRI Quality Control

All MRI scans were reviewed and reported by expert perinatal radiologists. The presence of any structural abnormalities was recorded, and image quality was scored on a scale from 1 to 4, based on the signal:noise ratio, presence of artifacts, field of view, and residual motion (4=high quality, 3=acceptable, 2=poor, 1=failed). Only subjects scoring 3 or 4 were included in the study, as previously described.^14^

### Cerebral Substrate Delivery Categorization

To explore the hypothesis that impaired cerebral substrate delivery plays a major role in the neurodevelopmental abnormalities associated with CHD, we divided the CHD cohort into 4 groups, according to the predicted level of substrate delivery to the developing fetal brain, based on the expected consequence of the underlying cardiac defect as described previously.^14^

In the absence of direct measurements of cerebral substrate delivery, cases were classified by a fetal cardiologist as described in^14^ according to the expected effect of the underlying cardiac defect on the delivery of oxygen and nutritional content of blood to the carotid arteries, and by extension, the brain. MRI‐derived fetal blood flows were used where available, as described in.^45^ For cases where information about the direction of blood flow at the aortic isthmus could not be derived from MRI, and was important for CHD categorization, it was extracted from the clinical fetal echocardiogram report, acquired as per routine clinical care. In cases where a diagnosis could potentially fit into more than 1 category, depending on severity or underlying hemodynamics, a combination of phase contrast (with metric optimized gating), fetal flow measurements,^45^ or contemporaneously acquired echocardiographic data were used to assign cases individually, following assessment of the data by a fetal cardiologist.^14^ Additional information about how each diagnosis was categorized can be found in Table S1.

The groups were as follows:

1. Substrate content of cerebral blood is expected to be normal
2. Substrate content of cerebral blood is expected to be mildly reduced (ie, some mixing of placental and fetal systemic venous blood).
3. Substrate content of cerebral blood is expected to be moderately reduced (ie, complete mixing of placental and fetal systemic venous blood)
4. Substrate content of cerebral blood is expected to be severely reduced (ie, complete reversal of normal placental streaming)

### Image Registration and Jacobian Determinant Calculation

Nonlinear deformation fields were calculated to transform the native subject T2 to the age‐matched template of the developing human connectome project fetal atlas (https://doi.gin.g‐node.org/10.12751/g‐node.ysgsy1/) using Advanced Normalization Tools symmetric diffeomorphic image registration.^46^ Warps were then concatenated between native T2, the age‐matched template, and a 30‐gestational week template space, which represents the median age of the cohort.^46^ The log Jacobian determinant was calculated on the concatenated warp, which represents the contraction and expansion of brain regions during image registration. In the resultant log Jacobian maps, higher log‐Jacobian values represent brain regions that contracted during image registration (ie, larger global and local brain volume), and smaller values represent smaller volume.^47^ The log Jacobian volumes for all subjects were concatenated to create a single 4‐dimensional file, which was used as the input for the ICA.^48^

### Independent Component Analysis of Jacobian Determinants

ICA is a data‐driven, blind source separation technique that extracts salient patterns embedded in the data; it reduces the dimensionality of neuroimaging data (from many thousands of individual voxels) by separating the multivariate signal into a maximally independent set of components.^49^ When applied in the spatial domain to structural imaging data (the log Jacobian determinants), ICA can detect coordinated growth between spatially separated brain regions, that is, SCNs, which are strongly associated with other clinical or demographic variables.^50^, ^51^, ^52^

A canonical ICA algorithm^48^ was used, implemented in Python using the nilearn package.^53^ The ICA algorithm transforms the input data into components (or SCNs) that represent an “unmixing” of the signal, such that the independent components have distributions that are non‐Gaussian. The optimal number of SCNs (n=40) for this case was chosen by surveying previous literature, and to balance robustness and interpretability.^39^, ^54^ When the ICA dimensionality was increased >40, visual inspection of components showed a division of cortical regions and splitting bilateral components into left/right lateralized areas.

The criteria for excluding components were (1) majority of the signal occurring in edge voxels, indicating misregistration (2) sparse, randomly distributed signal with low total area. For this data set, all components passed the exclusion threshold and were included in subsequent analyses.

To extract weights, or “modes,” for each network in each individual subject, FMRIB Software Library's (FSL) general linear model was applied to the component maps and the Jacobian input volumes.^55^, ^56^

### Application of the General Linear Model to Identify Covariates of Brain Structure

For each set of SCN weights, we fit a general linear model, with SCN weights as the dependent variable, to test the hypothesis that age, sex, and cerebral substrate delivery were significant predictors affecting the modes of variation across the cohort.

We fit either (1A) or (1B) depending on whether the relationship between the SCN weights and gestational age (GA) was linear or second order polynomial (determined by the Akaike information criterion^57^) (Figure S1).

(A) Modes~GA+sex+cerebral substrate delivery
  (B) Modes~GA+GA^2^+sex+cerebral substrate delivery

We then used a subset of the cohort, to explore the effect of cardiovascular physiology more specifically for each CHD diagnosis. We selected diagnosis categories with >10 subjects. These included tetralogy of Fallot (ToF) (n=13), TGA (n=22), right aortic arch (n=88), HLHS (n=25), double aortic arch (n=27), coarctation (+) (n=58), we also included fetuses with suspected coarctation prenatally who were not shown to have this condition in the neonatal period (“false positive” or coarctation (−)) (n=47). We included the coarctation (−) group in this analysis, as it has been shown previously that this group differs significantly from a healthy control population in terms of the distribution of the fetal circulation, for reasons that remain unclear.^45^ Because the purpose of this work was to investigate the effect of cerebral substrate delivery on brain maturation, this group was included in our analysis.

2 (A) Modes~GA+sex+CHD Diagnosis
  (B) Modes~GA+GA^2^+sex+CHD Diagnosis

We used false discovery rate correction for multiple comparisons (between 40 models, one for each SCN) to adjust the *P* values for all predictors before reviewing whether they were significant in each model.

## RESULTS

### Participants

A total of 435 subjects met the inclusion criteria, 67 were healthy controls (30 male) and 368 were diagnosed with CHD (182 male). All fetuses were scanned between 27‐ and 35‐weeks GA (Table 1).

**Table 1:** Cohort and Cerebral Substrate Delivery Group Demographic Information

Characteristics	Median GA (weeks)	IQR GA	Male sex
Congenital heart disease (n=368)	32.00	28.14–29.42	181
Control (n=67)	29.48	30.85–33.43	30
Cerebral substrate delivery group			
(1) Normal (n=232)	31.81	30.71–33.03	113
(2) Mildly reduced (n=67)	32.37	31.14–33.14	35
(3) Moderately reduced (n=46)	32.72	31.13–34.00	21
(4) Severely reduced (n=23)	33.12	32.14–34.71	12

GA indicates gestational age; and IQR, interquartile range.

A wide spectrum of CHD was represented in this cohort, including 40 different diagnoses (Table S2), which were categorized into 4 groups based on expected cerebral substrate delivery^14^: normal=232, mildly reduced=67, moderately reduced=46, severely reduced=23.

### Structural Covariance Networks in the Developing Fetal Brain With CHD

**Figure 1:**
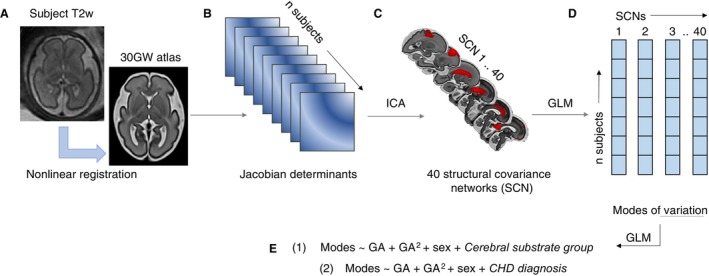
Methods pipeline to extract structural covariance networks. **A**, Reconstructed motion corrected T2w fetal magnetic resonance imaging is registered to the 30 GW atlas, using nonlinear registration. **B**, The log Jacobian of the warp is calculated. **C**, Jacobians are concatenated across all subjects and used as input to the canonical (ICA algorithm, to extract 40 SCNs. **D**, A (GLM is fit to the Jacobians and the SCNs, to extract an SCN weighting (or modes) for each subject. **E**, To examine the underlying sources of variation between modes for each SCN, 2 GLMs were fit, testing the effect of GA, GA^2^, sex, and either (1) cerebral substrate grouping or (2) CHD diagnosis. CHD indicates congenital heart disease; GA, gestational age; GLM, general linear model; GW, gestational week; ICA, independent component analysis; SCN, structural covariance network; and T2w, T2 weighted.

**Figure 2:**
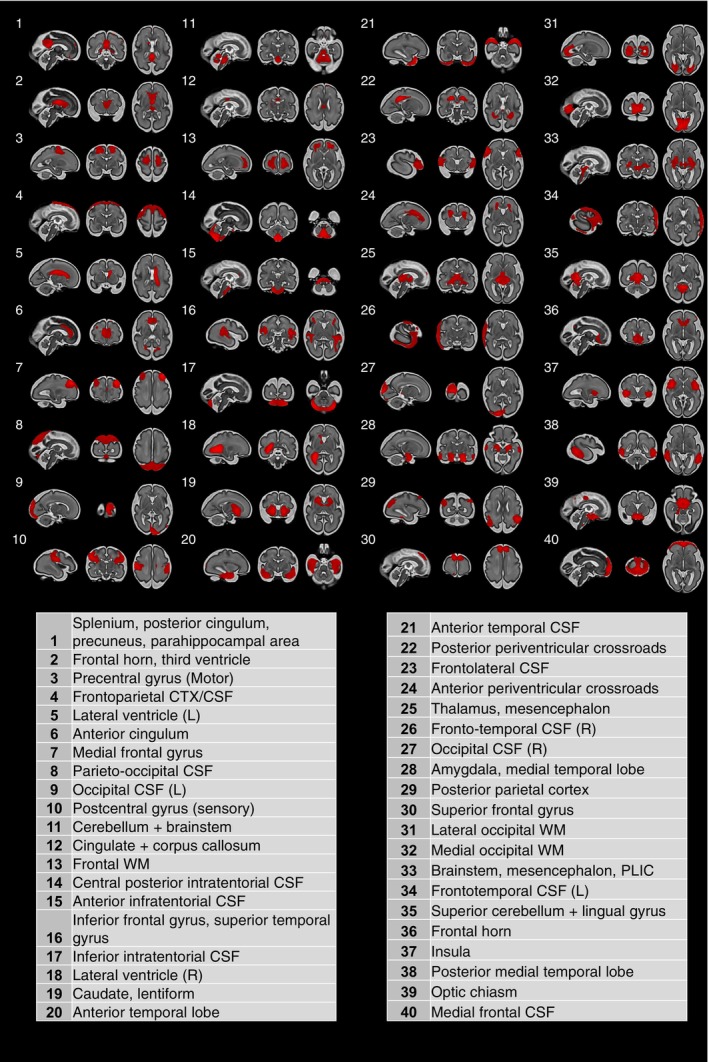
Structural covariance networks identified by ICA. A total of 40 independent structural covariance networks overlayed on a 30 gestational week fetal atlas. Table contains labels summarizing their neuroanatomy. CSF indicates cerebrospinal fluid; CTX, ; PLIC, posterior limb of the internal capsule; and WM, white matter.

As shown in Figure 1, we extracted 40 SCNs, each representing independent, coordinated structural development between brain regions. ICA was effective at extracting meaningful neuroanatomical structures, and labels were given to each SCN according to the corresponding anatomy (Figure 2). Almost all networks were either bilateral and symmetrical or had a complementary contralateral homolog component in the opposite hemisphere (Figure 2). All tissue types in the brain were represented, including deep gray matter, white matter, cortical gray matter, and cerebrospinal fluid (CSF). Certain SCNs were isolated to a specific tissue, and others included multiple tissue types. Charting the SCN modes against GA shows general maturational trends (Figure S1); however, there is considerable variability between subjects of the same age, suggesting that there are other significant sources of variation between individuals, such as the abnormal fetal hemodynamics, for a large proportion of this cohort.

### Structural Covariance Is Associated With Cerebral Substrate Delivery

We fitted a general linear model to each set of component weightings (also referred to as modes) to test whether age, sex, and cerebral substrate delivery were underlying sources of variation across the cohort. We found that 35 SCNs were significantly associated with GA, 9 with sex, and 14 with expected cerebral substrate delivery after false discovery rate correction for multiple comparisons between 40 independent components (ICs) (q<0.05) (Table 2). When we examined each substrate delivery group compared with controls, we found significant variation between controls and CHD where cerebral substrate delivery was expected to be normal, in 3 SCNs, including the left and right frontal temporal cortex/CSF networks, and frontoparietal cortex. These networks were also significantly different between controls and groups 2 and 3 (mildly and moderately reduced). In addition, there were differences between controls and mildly and moderately reduced groups in the medial frontal gyrus. The moderately reduced group were uniquely different from controls in the postcentral gyrus and optic chiasm, and different from the expected normal group in these networks. The moderately reduced group were also different from the expected normal group in the anterior cingulum, thalamus, and mesencephalon networks.

**Table 2:** Eighteen SCNs Where Cerebral Substrate Delivery and Congenital Heart DiseaseCHD Diagnosis Are Significant Predictors of Variance

SCN	Anatomy	% Variance explained	GA fit	GA significance	Sex	(1) SCN weights~GA+sex+cerebral substrate delivery group (FDR, q<0.05)										(2) SCN weights~GA+sex+CHD diagnosis (FDR, q<0.05)						
						Control vs 1	Control vs 2	Control vs 3	Control vs 4	1 vs 2	1 vs 3	1 vs 4	2 vs 3	2 vs 4	3 vs 4	Coarctation (−)	Coarctation (+)	DAA	HLHS	RAA	TGA	ToF
3	Precentral gyrus (motor)	1.74	0	0	0	0.857	0.109	0.187	0.739	0.011*^†^	0.077	0.533	0.674	0.025^*^	0.077	0.620	0.561	0.561	0.404	0.998	0.561	0.561
4	Frontoparietal cortex/CSF	4.27	1	1	0	0.001^*^^‡^	0.002^*^^‡^	0.001^*^^‡^	0.067	0.764	0.370	0.764	0.719	0.764	0.322	0.006*^‡^	0.014*^†^	0.001*^‡^	0.001*^‡^	0.008*^‡^	0.093	0.001*^‡^
5	Lateral ventricle (Left)	3.68	1	1	0	0.799	0.816	0.799	0.799	0.574	0.590	0.574	0.574	0.816	0.432	0.842	0.842	0.842	0.842	0.842	0.842	0.842
6	Anterior cingulum	1.61	2	1	0	0.974	0.798	0.167	0.565	0.556	0.022*^†^	0.385	0.288	0.599	0.518	0.870	0.589	0.589	0.083	0.870	0.524	0.524
7	Medial frontal gyrus	1.25	1	1	0	0.389	0.010^*^^‡^	0.018*^†^	0.599	0.010*^†^	0.029*^†^	0.910	0.900	0.085	0.127	0.964	0.028*^†^	0.993	0.056	0.339	0.964	0.424
9	Occipital cortex/CSF (Left)	1.60	1	1	0	0.762	0.010^*^^‡^	0.762	0.766	0.002	0.897	0.691	0.014^*^^†^	0.008*^‡^	0.762	0.745	0.320	0.818	0.818	0.818	0.818	0.012*^†^
10	Postcentral gyrus (sensory)	1.72	1	1	0	0.244	0.122	0.019*^†^	0.244	0.326	0.036*	0.513	0.262	0.879	0.265	0.519	0.377	0.492	0.037*^†^	0.519	0.377	0.816
11	Cerebellum+ brainstem	3.91	1	1	1	0.253	0.253	0.814	0.263	0.465	0.155	0.036^*^^‡^	0.082	0.020*^‡^	0.336	0.258	0.518	0.412	0.258	0.258	0.258	0.962
14	Cerebellum/intratentorial cortex/CSF	2.82	2	1	1	0.518	0.411	0.712	0.518	0.002^*^^‡^	0.216	0.170	0.250	0.697	0.712	0.796	0.832	0.832	0.832	0.832	0.796	0.407
16	Inferior frontal gyrus, superior temporal gyrus	1.35	1	1	0	0.553	0.704	0.263	0.104	0.700	0.454	0.144	0.377	0.128	0.292	0.879	0.879	0.102	0.015*^†^	0.343	0.102	0.343
25	Thalamus, mesencephalon	1.71	2	1	1	0.755	0.347	0.268	0.268	0.056	0.038*^‡^	0.038*^‡^	0.592	0.415	0.592	0.675	0.675	0.833	0.675	0.833	0.675	0.675
26	Frontotemporal cortex/CSF (Right)	5.12	1	1	0	0.025*^†^	0.025*^†^	0.025*^†^	0.274	0.397	0.397	0.730	0.926	0.457	0.443	0.189	0.424	0.424	0.057	0.057	0.424	0.990
27	Occipital cortex/CSF (Right)	1.62	1	1	0	0.127	0.237	0.122	0.311	0.925	0.560	0.925	0.563	0.973	0.438	0.089	0.074	0.530	0.016*^†^	0.119	0.530	0.929
32	Medial occipital white matter	2.46	2	1	0	0.095	0.095	0.013*	0.439	0.650	0.142	0.650	0.280	0.650	0.104	0.191	0.118	0.761	0.052	0.426	0.571	0.021*^†^
34	Frontotemporal cortex/CSF (Left)	5.26	1	1	0	0.024*^†^	0.024*^†^	0.024*^†^	0.933	0.406	0.406	0.235	0.793	0.111	0.088	0.370	0.370	0.370	0.143	0.095	0.833	0.044*^†^
37	Insula	1.54	2	1	0	0.352	0.352	0.352	0.117	0.632	0.632	0.238	0.931	0.423	0.562	0.221	0.733	0.362	0.072	0.420	0.048*^†^	0.048*^†^
39	Optic chiasm	1.99	1	1	0	0.826	0.826	0.048*^†^	0.826	0.360	0.001^*^^‡^	0.699	0.119	0.777	0.058	0.521	0.968	0.521	0.417	0.968	0.968	0.968
40	Medial frontal cortex/CSF	2.71	1	1	0	0.182	0.040*^†^	0.071	0.281	0.223	0.237	0.808	0.807	0.503	0.683	0.520	0.520	0.934	0.520	0.520	0.520	0.224

Adjusted *P* values after false discovery rate correction for multiple comparisons between components. Table also contains column of the proportion of total variance across the cohort explained by each SCN, whether there was a relationship with GA, (0=no relationship, 1=linear, or 2=second order polynomial), and if GA/sex were significant predictors of variance in the general linear model. CHD indicates congenital heart disease; CSF, cerebrospinal fluid; DAA, double aortic arch; FDR, false discovery rate; GA, gestational age; HLHS, hypoplastic left heart syndrome; RAA, right aortic arch; SCN, structural covariance network; TGA, transposition of the great arteries; and ToF, tetralogy of Fallot.

* Indicates significant comparisons.

† q<0.05.

‡ q<0.01.

There were no SCNs that have significantly different modes of variation between controls and the severely reduced cerebral substrate delivery category. This might be due to a relatively small sample of fetuses in this category (n=23), limiting our statistical power to detect differences in this group. However, we did find networks distinguishing the severely reduced group with CHD from the expected normal and mildly reduced groups. These networks included the precentral gyrus, occipital cortex/CSF, cerebellum and brainstem, and thalamus/mesencephalon. No significant differences were found between the moderately and severely reduced groups for any of the networks.

Six of the CHD‐sensitive networks included high proportions of CSF, encompassing both extra‐axial and infratentorial regions, suggesting altered cortical expansion of these areas. These components also explained the highest proportion of the total variance across the cohort.

**Figure 3:**
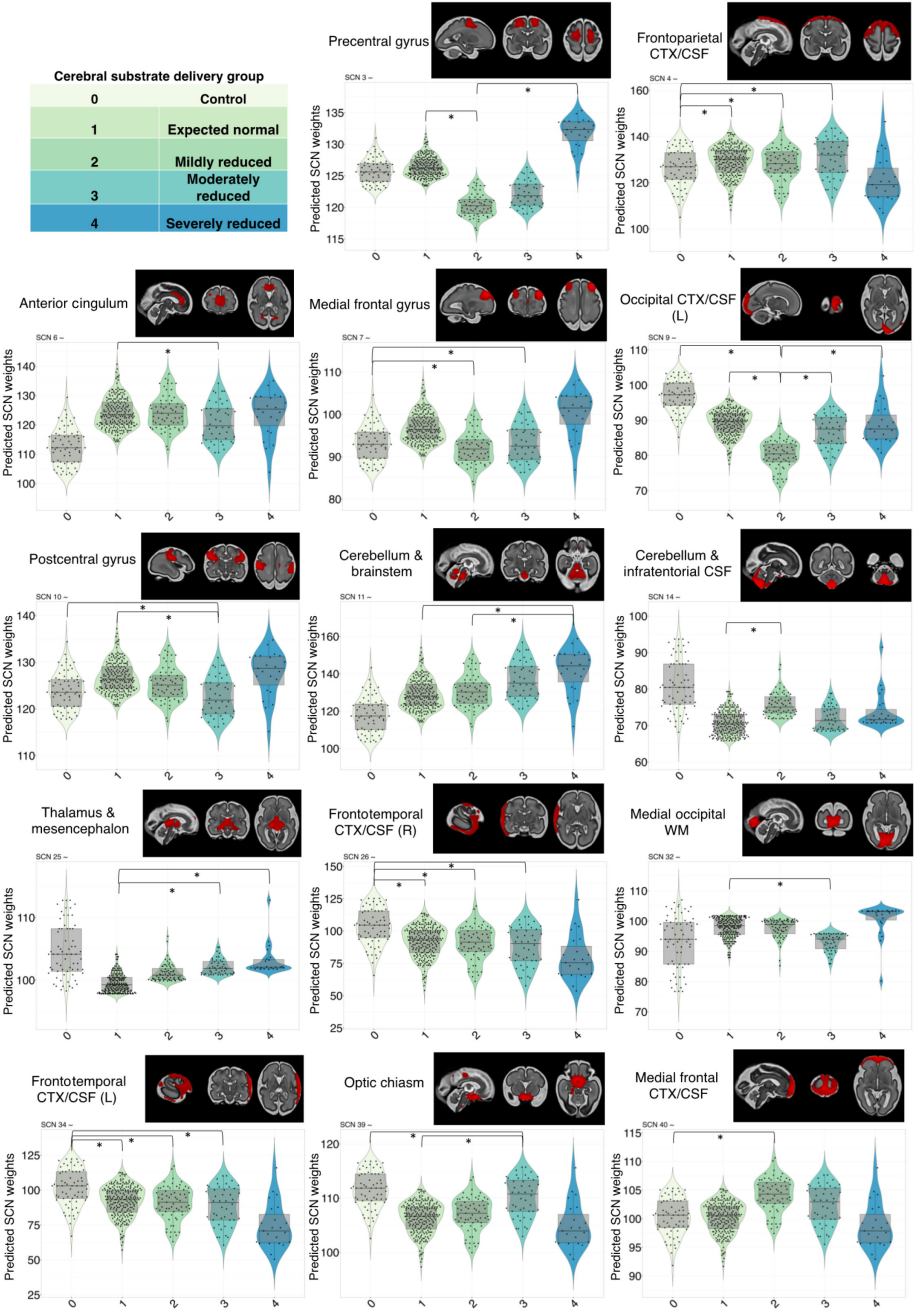
Structural covariance explained by cerebral substrate delivery. For each significant component, the general linear model predicted modes are shown (when accounting for gestational age, sex, and cerebral substrate delivery group), arranged along the *x* axis by cerebral substrate delivery groups (key shown in top left corner). (*) denotes significant difference between groups after correcting for multiple comparisons using false discovery rate (q<0.05). CSF indicates cerebrospinal fluid; CTX, SCN, structural covariance network; and WM, white matter.

We created violin plots to show the model‐fitted difference between groups, when accounting for age and sex, for components where there was a significant effect of expected cerebral substrate delivery (Figure 3). This highlighted the networks for which there was a gradient or dose‐dependent effect of cerebral substrate delivery, such as both left and right frontotemporal cortex/CSF networks and the cerebellum.

### The Effect of Specific CHD Diagnoses on Structural Brain Maturation

Given the differences in structural covariance across the brain between CHD cerebral substrate groups, we carried out a sensitivity analysis in a subset of the cohort to explore whether specific CHD diagnoses were predictors of variation (Table 2). In this way, we could test the hypothesis that specific cardiac defects have unique and distinct effects on structural brain maturation. We used a subset of the cohort (n=238), including only CHD diagnosis categories with >10 subjects (see Methods). We fit a general linear model with SCN weights as the dependent variable and tested for a combined effect of age, age squared, sex, and CHD diagnosis. There were 9 different SCNs where at least 1 of the CHD diagnosis categories explained a significant amount of the variation between individuals. The summary of SCNs that were significant for each diagnosis category after false discovery rate correction (q<0.05) are shown in Figure 3.

We found the frontoparietal network was significantly different between controls and all CHD diagnoses except for TGA (Table 2). We also found certain SCNs, where only 1 CHD diagnosis group was significantly different from controls. Coarctation (+) explained a significant proportion of the variation in the network corresponding to the medial frontal gyrus. HLHS explained a significant proportion of the variance in the postcentral gyrus, inferior frontal and superior temporal gyrus, and occipital cortex/CSF. Fetuses with ToF were uniquely different from controls in the frontotemporal cortex, medial occipital white matter, and occipital cortex. Both ToF and TGA were significant predictors of variance in the insula network.

## DISCUSSION

In this study we applied an unsupervised, data‐driven analysis technique, ICA, to capture the dynamic T2w contrast changes across the fetal brain in space and time, summarizing them into an interpretable set of anatomically meaningful networks, or SCNs.^58^ The networks we derived correspond to recognizable structures in the fetal brain, including regions that are developmentally critical but transiently present in the fetal period, such as anterior and posterior periventricular crossroads. We examined whether GA, sex, cerebral substrate delivery, and CHD diagnosis are significant factors determining the variation between individuals in structural brain maturation. We used the networks to stratify the large heterogenous cohort of different CHD cases, identifying variation across the brain according to cerebral substrate delivery and specific CHD diagnosis. Of the 40 SCNs we derived, we found a significant effect of cerebral substrate delivery or CHD diagnosis in 18 of them.

### The Maturation of Specific Networks Is Vulnerable to the Effects of Reduced Cerebral Substrate Delivery

Previous work has demonstrated that hemodynamic alterations in fetuses with CHD contribute to abnormalities in brain development.^9^, ^19^ Volumetric differences correlate with cerebral substrate delivery in the third trimester,^14^, ^20^ which represents a critical window of accelerated fetal brain growth and increased cerebral metabolic demands. A reasonable hypothesis to explain these findings is that reduced cerebral substrate delivery, secondary to altered fetal cardiovascular physiology, is a driving factor in the emergence of structural brain differences in CHD.^29^ In this study, we identified networks of brain regions vulnerable to the effects of altered cerebral substrate delivery which can be broadly grouped as follows: (1) gyral areas, pre‐ and postcentral, medial frontal; (2) thalamus and brainstem; (3) cerebellum and infratentorial CSF; and (4) cortical/CSF regions, frontoparietal, and frontotemporal and occipital cortex/CSF. The sensitivity of these particular regions is congruent with results of other studies, also noting the most significant volumetric changes in CHD in the frontal lobe and the brainstem,^17^ impaired expansion of higher cortical areas,^59^ altered patterning and delayed maturation of cortical folds,^10^, ^13^, ^17^, ^60^ abnormal cerebellar development,^61^ and enlarged CSF spaces.^9^, ^11^, ^62^, ^63^, ^64^ Because the Jacobian determinant represents the contraction and expansion of brain regions during image registration, the significance of networks in CSF spaces reflects an altered ratio of brain tissue:fluid in these areas, in the context of the dynamic and fast paced fetal brain growth. Studies across multiple different cohorts, spanning a large GA range, between 20 weeks GA and term, have reported increased extra‐axial CSF spaces in the fetal brain with CHD^9^, ^11^, ^62^, ^63^, ^64^ which has been interpreted as a general marker of delayed brain development in this population.^11^

### Cerebral Substrate Delivery Explains Some But Not All of the Variance in CHD

We observed differences between healthy controls and fetuses with CHD where cerebral substrate delivery is expected to be normal, in frontoparietal, frontotemporal, and occipital cortical/CSF networks, suggesting extrinsic factors to this analysis, such as genetic and environmental factors, also mediate the differences in early brain growth. Furthermore, when we explored specific CHD diagnoses in a subset of the cohort, we recapitulated this result, finding coarctation (−), right aortic arch, and double aortic arch subtypes predicted the variance in the same frontoparietal cortex/CSF networks. These diagnoses are considered milder forms of CHD where cerebral substrate delivery is expected to be normal.^29^ An alternative explanation for the difference in brain development is genetic variation among the milder phenotypes, which has not been accounted for in this analysis. Previous work has highlighted shared underlying genetic pathways between the heart and brain that may account for the phenotype of altered structural brain development in CHD.^65^, ^66^ Genetic abnormalities are also highly prevalent in the CHD population,^22^ and protein‐damaging de novo mutations have been identified for genes highly expressed in both the developing heart and brain.^67^, ^68^

The residual variation in brain development between individuals in this work may also be mediated by differences in placental function.^24^, ^25^ The parallel development between fetal and maternal organs, and the shared genetic and developmental pathways between the heart, brain, and placenta, are emerging as important contributing factors to the vulnerability of this patient group.^69^, ^70^, ^71^, ^72^ Placental imaging studies have highlighted a critical relationship between placental size and overall fetal growth, in both healthy and at‐risk populations, suggesting that over a third of birth weight variation is due to placental weight.^73^ A growing body of evidence implicates abnormal placental structure and function in CHD pathology,^24^, ^69^, ^70^, ^71^, ^72^ and future work would benefit from investigating these effects on the structural brain.

### Specific Diagnoses Were Better Predictors of Variance Than Others

When examining the differences between CHD diagnoses, HLHS and ToF were the most significant predictors of variance across different networks, in 4 and 5 SCNs respectively. We found a unique effect of HLHS diagnosis on 3 structural networks, including the postcentral gyrus, inferior frontal and superior temporal gyrus, and occipital cortical/CSF. In fetuses with ToF, the medial occipital white matter, the insula, and the frontotemporal and occipital cortex were also significantly different from healthy controls. Previous studies have also observed a severe effect on brain development in fetuses and neonates diagnosed with either ToF^62^ or HLHS.^74^ One study reported significantly reduced cardiac output in fetuses with HLHS and a dose‐dependent effect on delayed brain maturation.^19^ Our analysis also highlighted the insula network as significantly different for fetuses with ToF or TGA compared with controls, in accordance with previous work noting delayed opercular development in term infants with complex CHD.^17^, ^74^, ^75^, ^76^ Operculation of the insula is usually complete at term,^77^ although an open operculum and exposed insular cortex have been associated with neurodevelopmental delays in CHD and more broadly in other patient populations.^78^, ^79^, ^80^, ^81^

Interestingly, TGA was not significantly different from controls for any of the networks except the insula. The severely reduced cerebral substrate delivery group is composed mostly of fetuses with TGA, and we do not observe any distinctly different networks for this category. This group deviate from our hypothesis, highlighting that there is not necessarily a dose‐dependent effect of expected cerebral substrate delivery on brain morphometry. Although our previous work has shown that whole brain volume is reduced in this cohort of fetuses with TGA,^14^ and is disproportionately smaller than the volume of the fetal body,^14^, ^82^ our results suggest that once accounting for global reductions, the local effects across the brain are minimal. It is plausible that the smaller global brain size for this subtype is accompanied by proportionally smaller brain structures, and therefore we do not detect abnormalities in the coordinated growth of specific structural networks.

### CHD‐Sensitive Networks in Frontal Cortical Regions

Specific networks emerged as being affected by both cerebral substrate delivery and CHD diagnosis categories. These included the frontoparietal, frontotemporal, and occipital cortex. In the fetal period, these networks likely represent altered volumes of CSF, in conjunction with the areal expansion and gyrification of the cortices. Previous work using a porcine model explored the developmental processes effected by both transient and chronic disturbances in fetal oxygen delivery,^83^, ^84^, ^85^ resolving that this may provide a mechanistic explanation for the effect of CHD on the development of higher order cortical areas. They established that inducing chronic hypoxic exposure decreases neuronal proliferation, migration, interneuron populations and overall volume in the insula and prefrontal cortices.^85^ In the same study, a parallel analysis of postmortem brain tissue from infants with complex CHD revealed less mature astrocytic processes, and a depletion of neuroblasts within the subventricular zone in frontal areas.^85^ The authors speculate that this cellular phenotype may propagate to the level of neuronal circuits, creating an excitatory/inhibitory imbalance in the developing cortex in CHD.^85^ Excitatory/inhibitory imbalance has been investigated across a wide spectrum of intellectual and behavioral disabilities, potentially explaining why children with CHD show deficits in cognitive domains associated with higher order cortices.^59^

### White Matter Dominated Networks Were Largely Not Affected by CHD Diagnosis

Many white matter networks were extracted by this analysis, reflecting the growth of key white matter structures and development of fiber connectivity over the third trimester,^86^, ^87^, ^88^ including both the anterior and posterior periventricular crossroads and the corpus callosum/cingulum components. However, in most of these networks we did not detect significant differences between CHD and the control population. Previous studies characterizing white matter development in utero in fetuses with CHD^89^, ^90^ show smaller white matter volumes, which may just reflect the overall reduction in brain size in this group. Diffusion weighted imaging may be more appropriate to elucidate any aberrant white matter development in CHD, as changes can be examined at the microstructural level. Previous studies using in utero diffusion weighted imaging in CHD, found certain localized group level differences in the corpus callosum,^91^ frontal white matter, and thalamus.^92^ However, for a variety of brain regions no differences were found in diffusion metrics between patients and controls.^92^, ^93^ Examining age‐specific differences between CHD and controls would improve our understanding of whether white matter development is abnormal in this population, as diffusion metrics vary nonlinearly with GA,^88^ and dynamic changes occur between the fetal brain layers that are precursors to white matter.^94^

### CONCLUSIONS

Overall, this analysis framework offers an alternative approach to studying a heterogenous patient group, capturing variation associated with age, cerebral substrate delivery, and CHD diagnosis. This work contributes to building a more comprehensive understanding of brain development in CHD before birth, in utero, highlighting regions that maturing differently in this vulnerable population. Our unconstrained data‐driven approach identified the same vulnerable brain regions as previous work,^9^, ^10^, ^11^, ^17^, ^61^, ^62^, ^63^, ^74^, ^75^, ^76^, ^82^, ^95^ which was reliant on a priori segmentations. The reproducibility of this result supports that these findings are a meaningful reflection of biological differences between the patient and control group.

The results of this study demonstrate the potential for neuroimaging data in the fetal period to explore the unique effects of different CHD diagnoses on in utero structural brain development. They also suggest that the fetal period represents a critical window for targeted interventions such as maternal hyperoxygenation,^31^, ^96^ which aims to improve cerebral oxygen delivery in vulnerable fetuses. Other neuroprotective interventions aiming to reduce brain injury such as allopurinol^97^ or progesterone^98^ are also under investigation and may help correct the altered trajectory of impaired early brain growth seen in CHD.

### Limitations

There are some important limitations to note with this study. First, the distribution of GAs between controls and fetuses with CHD was different. Although our model included GA, this may still result in some false positive or false negative significant networks. We were unable to discern the delivery of specific substrates, or measure substrate delivery quantitatively for every fetus. Differences in cerebral vessel density may contribute to altered structural brain development in CHD. Specifically, capillary density levels in certain brain regions may not stabilize until years after birth. Future work would benefit from measuring cerebral vessel density or cerebral perfusion to directly test this.

The cerebral substrate delivery groups also had uneven sample sizes, with a much higher proportion of subjects in the expected normal group than any other group. This gave us more statistical power to detect differences between group 1 and controls compared with other groups, which may have led to some false negatives. Similarly for CHD diagnoses, more severe subtypes (TGA, ToF, and HLHS) were less common, therefore there was less statistical power to detect differences between these categories. All imaging data were acquired on the same scanner, in the same hospital catchment area in central London, and despite the socioeconomic and racial diversity of London, our result may not be generalizable to other populations.

## Sources of Funding

This research was funded by the Medical Research Council UK (MR/L011530/1 and MR/V002465/1) and the Wellcome Trust (102431). This research was supported by core funding from the Wellcome/Engineering and Physical Sciences Research Council Centre for Medical Engineering (WT203148/Z/16/Z), and by the National Institute for Health Research Biomedical Research Centre based at Guy's and St Thomas’ National Health Service Foundation Trust and King's College London. The views expressed are those of the authors and not necessarily those of the National Health Service, the National Institute for Health Research, or the Department of Health.

## Disclosures

None.

## Supporting information

Tables S1–S2
Figure S1

## References

[1] Dolk H , Loane M , Garne E ; a European Surveillance of Congenital Anomalies (EUROCAT) Working Group . Congenital heart defects in Europe: prevalence and perinatal mortality, 2000 to 2005. Circulation. 2011;123:841–849. doi: 10.1161/CIRCULATIONAHA.110.958405 21321151

[2] Bouma BJ , Mulder BJM . Changing landscape of congenital heart disease. Circ Res. 2017;120:908–922. doi: 10.1161/CIRCRESAHA.116.309302 28302739

[3] Dellborg M , Giang KW , Eriksson P , Liden H , Fedchenko M , Ahnfelt A , Rosengren A , Mandalenakis Z . Adults with congenital heart disease: trends in event‐free survival past middle age. Circulation. 2023;147:930–938. doi: 10.1161/CIRCULATIONAHA.122.060834 36571845 PMC10022672

[4] Latal B . Neurodevelopmental outcomes of the child with congenital heart disease. Clin Perinatol. 2016;43:173–185. doi: 10.1016/j.clp.2015.11.012 26876129

[5] Newburger JW , Sleeper LA , Bellinger DC , Goldberg CS , Tabbutt S , Lu M , Mussatto KA , Williams IA , Gustafson KE , Mital S , et al. Early developmental outcome in children with hypoplastic left heart syndrome and related anomalies: the single ventricle reconstruction trial. Circulation. 2012;125:2081–2091. doi: 10.1161/CIRCULATIONAHA.111.064113 22456475 PMC3341507

[6] Naef N , Liamlahi R , Beck I , Bernet V , Dave H , Knirsch W , Latal B . Neurodevelopmental profiles of children with congenital heart disease at school age. J Pediatr. 2017;188:75–81. doi: 10.1016/j.jpeds.2017.05.073 28709631

[7] Marelli A , Miller SP , Marino BS , Jefferson AL , Newburger JW . Brain in congenital heart disease across the lifespan: the cumulative burden of injury. Circulation. 2016;133:1951–1962. doi: 10.1161/CIRCULATIONAHA.115.019881 27185022 PMC5519142

[8] Bellinger DC , Wypij D , Rivkin MJ , DeMaso DR , Robertson RL , Dunbar‐Masterson C , Rappaport LA , Wernovsky G , Jonas RA , Newburger JW . Adolescents with d‐transposition of the great arteries corrected with the arterial switch procedure: neuropsychological assessment and structural brain imaging. Circulation. 2011;124:1361–1369. doi: 10.1161/CIRCULATIONAHA.111.026963 21875911 PMC3217719

[9] Limperopoulos C , Tworetzky W , McElhinney DB , Newburger JW , Brown DW , Robertson RL , Guizard N , McGrath E , Geva J , Annese D , et al. Brain volume and metabolism in fetuses with congenital heart disease: evaluation with quantitative magnetic resonance imaging and spectroscopy. Circulation. 2010;121:26–33. doi: 10.1161/CIRCULATIONAHA.109.865568 20026783 PMC2819908

[10] Clouchoux C , Du Plessis AJ , Bouyssi‐Kobar M , Tworetzky W , McElhinney DB , Brown DW , Gholipour A , Kudelski D , Warfield SK , McCarter RJ , et al. Delayed cortical development in fetuses with complex congenital heart disease. Cereb Cortex. 2013;23:2932–2943. doi: 10.1093/cercor/bhs281 22977063

[11] Brossard‐Racine M , Du Plessis AJ , Vezina G , Robertson R , Bulas D , Evangelou IE , Donofrio M , Freeman D , Limperopoulos C . Prevalence and Spectrum of in utero structural brain abnormalities in fetuses with complex congenital heart disease. Am J Neuroradiol. 2014;35:1593–1599. doi: 10.3174/ajnr.A3903 24651820 PMC7964442

[12] Ren J , Zhu M , Dong S . Three‐dimensional volumetric magnetic resonance imaging detects early alterations of the brain growth in fetuses with congenital heart disease. J Magn Reson Imaging. 2021;54:263–272. doi: 10.1002/jmri.27526 33559371

[13] Dovjak GO , Zalewski T , Seidl‐Mlczoch E , Ulm PA , Berger‐Kulemann V , Weber M , Prayer D , Kasprian GJ , Ulm B . Abnormal Extracardiac development in fetuses with congenital heart disease. J Am Coll Cardiol. 2021;78:2312–2322. doi: 10.1016/j.jacc.2021.09.1358 34857093

[14] Cromb D , Uus A , Van Poppel MPM , Steinweg JK , Bonthrone AF , Maggioni A , Cawley P , Egloff A , Kyriakopolous V , Matthew J , et al. Total and regional brain volumes in fetuses with congenital heart disease. J Magn Reson Imaging. 2023;60:jmri.29078. doi: 10.1002/jmri.29078 PMC7616254 37846811

[15] Rollins CK , Ortinau CM , Stopp C , Friedman KG , Tworetzky W , Gagoski B , Velasco‐Annis C , Afacan O , Vasung L , Beaute JI , et al. Regional brain growth trajectories in fetuses with congenital heart disease. Ann Neurol. 2021;89:143–157. doi: 10.1002/ana.25940 33084086 PMC7970443

[16] Wu Y , Lu Y‐C , Kapse K , Jacobs M , Andescavage N , Donofrio MT , Lopez C , Quistorff JL , Vezina G , Krishnan A , et al. In utero MRI identifies impaired second trimester subplate growth in fetuses with congenital heart disease. Cereb Cortex. 2022;32:2858–2867. doi: 10.1093/cercor/bhab386 34882775 PMC9247421

[17] Ortinau CM , Rollins CK , Gholipour A , Yun HJ , Marshall M , Gagoski B , Afacan O , Friedman K , Tworetzky W , Warfield SK , et al. Early‐emerging Sulcal patterns are atypical in fetuses with congenital heart disease. Cereb Cortex. 2019;29:3605–3616. doi: 10.1093/cercor/bhy235 30272144 PMC6644862

[18] Jaimes C , Machado‐Rivas F , Afacan O , Khan S , Marami B , Ortinau CM , Rollins CK , Velasco‐Annis C , Warfield SK , Gholipour A . In vivo characterization of emerging white matter microstructure in the fetal brain in the third trimester. Hum Brain Mapp. 2020;41:3177–3185. doi: 10.1002/hbm.25006 32374063 PMC7375105

[19] Sun L , Macgowan CK , Sled JG , Yoo S‐J , Manlhiot C , Porayette P , Grosse‐Wortmann L , Jaeggi E , McCrindle BW , Kingdom J , et al. Reduced fetal cerebral oxygen consumption is associated with smaller brain size in fetuses with congenital heart disease. Circulation. 2015;131:1313–1323. doi: 10.1161/CIRCULATIONAHA.114.013051 25762062 PMC4398654

[20] Peyvandi S , Xu D , Wang Y , Hogan W , Moon‐Grady A , Barkovich AJ , Glenn O , McQuillen P , Liu J . Fetal cerebral oxygenation is impaired in congenital heart disease and shows variable response to maternal Hyperoxia. J Am Heart Assoc. 2021;10:e018777. doi: 10.1161/JAHA.120.018777 33345557 PMC7955474

[21] Sun L , Van Amerom JFP , Marini D , Portnoy S , Lee F‐T , Saini BS , Lim JM , Aguet J , Jaeggi E , Kingdom JC , et al. MRI characterization of hemodynamic patterns of human fetuses with cyanotic congenital heart disease. Ultrasound Obstet Gynecol. 2021;58:824–836. doi: 10.1002/uog.23707 34097323

[22] Blue GM , Kirk EP , Giannoulatou E , Sholler GF , Dunwoodie SL , Harvey RP , Winlaw DS . Advances in the genetics of congenital heart disease. J Am Coll Cardiol. 2017;69:859–870. doi: 10.1016/j.jacc.2016.11.060 28209227

[23] Maleyeff L , Newburger JW , Wypij D , Thomas NH , Anagnoustou E , Brueckner M , Chung WK , Cleveland J , Cunningham S , Gelb BD , et al. Association of genetic and sulcal traits with executive function in congenital heart disease. Ann Clin Transl Neurol. 2024;11:278–290. doi: 10.1002/acn3.51950 38009418 PMC10863927

[24] Cromb D , Slator PJ , Hall M , Price A , Alexander DC , Counsell SJ , Hutter J . Advanced magnetic resonance imaging detects altered placental development in pregnancies affected by congenital heart disease. Sci Rep. 2024;14:12357. doi: 10.1038/s41598-024-63087-8 38811636 PMC11136986

[25] Leon RL , Sharma K , Mir IN , Herrera CL , Brown SL , Spong CY , Chalak LF . Placental vascular malperfusion lesions in fetal congenital heart disease. Am J Obstet Gynecol. 2022;227:620.e1–620.e8. doi: 10.1016/j.ajog.2022.05.038 PMC13217299 35609643

[26] Rychik J , Donaghue DD , Levy S , Fajardo C , Combs J , Zhang X , Szwast A , Diamond GS . Maternal psychological stress after prenatal diagnosis of congenital heart disease. J Pediatr. 2013;162:302–307.e1. doi: 10.1016/j.jpeds.2012.07.023 22974576

[27] Wu Y , Kapse K , Jacobs M , Niforatos‐Andescavage N , Donofrio MT , Krishnan A , Vezina G , Wessel D , du Plessis A , Limperopoulos C . Association of maternal psychological distress with in utero brain development in fetuses with congenital heart disease. JAMA Pediatr. 2020;174:e195316. doi: 10.1001/jamapediatrics.2019.5316 31930365 PMC6990726

[28] Sadhwani A , Wypij D , Rofeberg V , Gholipour A , Mittleman M , Rohde J , Velasco‐Annis C , Calderon J , Friedman KG , Tworetzky W , et al. Fetal brain volume predicts neurodevelopment in congenital heart disease. Circulation. 2022;145:1108–1119. doi: 10.1161/CIRCULATIONAHA.121.056305 35143287 PMC9007882

[29] Rudolph AM . Congenital cardiovascular malformations and the fetal circulation. Arch Dis Child Fetal Neonatal Ed. 2010;95:F132–F136. doi: 10.1136/adc.2007.128777 19321508

[30] Donofrio MT , Bremer YA , Schieken RM , Gennings C , Morton LD , Eidem BW , Cetta F , Falkensammer CB , Huhta JC , Kleinman CS . Autoregulation of cerebral blood flow in fetuses with congenital heart disease: the brain sparing effect. Pediatr Cardiol. 2003;24:436–443. doi: 10.1007/s00246-002-0404-0 14627309

[31] Lee F‐T , Seed M , Sun L , Marini D . Fetal brain issues in congenital heart disease. Transl Pediatr. 2021;10:2182–2196. doi: 10.21037/tp-20-224 34584890 PMC8429876

[32] Studholme C , Cardenas V , Schuff N , Rosen H , Miller B , Weiner M . Detecting spatially consistent structural differences in Alzheimer's and Fronto temporal dementia using deformation morphometry. In: Niessen WJ , Viergever MA , editors. Medical Image Computing and Computer‐Assisted Intervention – MICCAI 2001. Springer Berlin Heidelberg; 2001: 41–48. 10.1007/3-540-45468-3_6

[33] Thompson PM , Giedd JN , Woods RP , MacDonald D , Evans AC , Toga AW . Growth patterns in the developing brain detected by using continuum mechanical tensor maps. Nature. 2000;404:190–193. doi: 10.1038/35004593 10724172

[34] Geng X , Li G , Lu Z , Gao W , Wang L , Shen D , Zhu H , Gilmore JH . Structural and maturational covariance in early childhood brain development. Cereb Cortex. 2017;27:1795–1807. doi: 10.1093/cercor/bhw022 26874184 PMC6059236

[35] Seeley WW , Crawford RK , Zhou J , Miller BL , Greicius MD . Neurodegenerative diseases target large‐scale human brain networks. Neuron. 2009;62:42–52. doi: 10.1016/j.neuron.2009.03.024 19376066 PMC2691647

[36] Li X , Pu F , Fan Y , Niu H , Li S , Li D . Age‐related changes in brain structural covariance networks. Front Hum Neurosci. 2013;7:98. doi: 10.3389/fnhum.2013.00098/abstract 23532684 PMC3607831

[37] Spreng RN , Turner GR . Structural covariance of the default network in healthy and pathological aging. J Neurosci. 2013;33:15226–15234. doi: 10.1523/JNEUROSCI.2261-13.2013 24048852 PMC3776065

[38] Fenchel D , Dimitrova R , Seidlitz J , Robinson EC , Batalle D , Hutter J , Christiaens D , Pietsch M , Brandon J , Hughes EJ , et al. Development of microstructural and morphological cortical profiles in the neonatal brain. Cereb Cortex. 2020;30:5767–5779. doi: 10.1093/cercor/bhaa150 32537627 PMC7673474

[39] Vanes LD , Hadaya L , Kanel D , Falconer S , Ball G , Batalle D , Counsell SJ , Edwards AD , Nosarti C . Associations between neonatal brain structure, the home environment, and childhood outcomes following very preterm birth. Biol Psychiatry Glob Open Sci. 2021;1:146–155. doi: 10.1016/j.bpsgos.2021.05.002 34471914 PMC8367847

[40] Bassett DS , Bullmore E , Verchinski BA , Mattay VS , Weinberger DR , Meyer‐Lindenberg A . Hierarchical organization of human cortical networks in health and schizophrenia. J Neurosci. 2008;28:9239–9248. doi: 10.1523/JNEUROSCI.1929-08.2008 18784304 PMC2878961

[41] Heinze K , Reniers RLEP , Nelson B , Yung AR , Lin A , Harrison BJ , Pantelis C , Velakoulis D , McGorry PD , Wood SJ . Discrete alterations of brain network structural covariance in individuals at ultra‐high risk for psychosis. Biol Psychiatry. 2015;77:989–996. doi: 10.1016/j.biopsych.2014.10.023 25524754

[42] He Y , Chen Z , Evans A . Structural insights into aberrant topological patterns of large‐scale cortical networks in Alzheimer's disease. J Neurosci. 2008;28:4756–4766. doi: 10.1523/JNEUROSCI.0141-08.2008 18448652 PMC6670444

[43] Kuklisova‐Murgasova M , Quaghebeur G , Rutherford MA , Hajnal JV , Schnabel JA . Reconstruction of fetal brain MRI with intensity matching and complete outlier removal. Med Image Anal. 2012;16:1550–1564. doi: 10.1016/j.media.2012.07.004 22939612 PMC4067058

[44] Uus A , Grigorescu I , Van Poppel M , Hughes E , Steinweg J , Roberts T , Lloyd D , Pushparajah K , Deprez M . 3D UNet with GAN discriminator for robust localisation of the fetal brain and trunk in MRI with partial coverage of the fetal body. Bioengineering. 2021:bioRxiv 2021.06.23.449574. doi: 10.1101/2021.06.23.449574

[45] Lloyd DFA , Van Poppel MPM , Pushparajah K , Vigneswaran TV , Zidere V , Steinweg J , Van Amerom JFP , Roberts TA , Schulz A , Charakida M , et al. Analysis of 3‐dimensional arch anatomy, vascular flow, and postnatal outcome in cases of suspected Coarctation of the aorta using fetal cardiac magnetic resonance imaging. Circ Cardiovasc Imaging. 2021;14:e012411. doi: 10.1161/CIRCIMAGING.121.012411 34187165 PMC8300852

[46] Avants B , Epstein C , Grossman M , Gee J . Symmetric diffeomorphic image registration with cross‐correlation: evaluating automated labeling of elderly and neurodegenerative brain. Med Image Anal. 2008;12:26–41. doi: 10.1016/j.media.2007.06.004 17659998 PMC2276735

[47] Avants B , Gee JC . Geodesic estimation for large deformation anatomical shape averaging and interpolation. NeuroImage. 2004;23:S139–S150. doi: 10.1016/j.neuroimage.2004.07.010 15501083

[48] Varoquaux G , Sadaghiani S , Pinel P , Kleinschmidt A , Poline JB , Thirion B . A group model for stable multi‐subject ICA on fMRI datasets. NeuroImage. 2010;51:288–299. doi: 10.1016/j.neuroimage.2010.02.010 20153834

[49] Jutten C , Herault J . Blind separation of sources, part I: an adaptive algorithm based on neuromimetic architecture. Signal Process. 1991;24:1–10. doi: 10.1016/0165-1684(91)90079-X

[50] O'Muircheartaigh J , Dean DC , Ginestet CE , Walker L , Waskiewicz N , Lehman K , Dirks H , Piryatinsky I , Deoni SCL . White matter development and early cognition in babies and toddlers. Hum Brain Mapp. 2014;35:4475–4487. doi: 10.1002/hbm.22488 24578096 PMC4336562

[51] Douaud G , Groves AR , Tamnes CK , Westlye LT , Duff EP , Engvig A , Walhovd KB , James A , Gass A , Monsch AU , et al. A common brain network links development, aging, and vulnerability to disease. Proc Natl Acad Sci USA. 2014;111:17648–17653. doi: 10.1073/pnas.1410378111 25422429 PMC4267352

[52] Llera A , Wolfers T , Mulders P , Beckmann CF . Inter‐individual differences in human brain structure and morphology link to variation in demographics and behavior. eLife. 2019;8:e44443. doi: 10.7554/eLife.44443 31268418 PMC6663467

[53] Abraham A , Pedregosa F , Eickenberg M , Gervais P , Mueller A , Kossaifi J , Gramfort A , Thirion B , Varoquaux G . Machine learning for neuroimaging with scikit‐learn. Front Neuroinformatics. 2014;8:14. doi: 10.3389/fninf.2014.0001 PMC3930868 24600388

[54] Eyre M , Fitzgibbon SP , Ciarrusta J , Cordero‐Grande L , Price AN , Poppe T , Schuh A , Hughes E , O'Keeffe C , Brandon J , et al. The developing human connectome project: typical and disrupted perinatal functional connectivity. Brain. 2021;144:2199–2213. doi: 10.1093/brain/awab118 33734321 PMC8370420

[55] Winkler AM , Ridgway GR , Webster MA , Smith SM , Nichols TE . Permutation inference for the general linear model. NeuroImage. 2014;92:381–397. doi: 10.1016/j.neuroimage.2014.01.060 24530839 PMC4010955

[56] Anderson MJ , Robinson J . Permutation tests for linear models. Aust N Z J Stat. 2001;43:75–88. doi: 10.1111/1467-842X.00156

[57] Akaike H . A new look at the statistical model identification. IEEE Trans Autom Control. 1974;19:716–723. doi: 10.1109/TAC.1974.1100705

[58] Comon P . Independent component analysis: a new concept? Signal Process. 1994;36:287–314. doi: 10.1016/0165-1684(94)90029-9

[59] Leonetti C , Back SA , Gallo V , Ishibashi N . Cortical dysmaturation in congenital heart disease. Trends Neurosci. 2019;42:192–204. doi: 10.1016/j.tins.2018.12.003 30616953 PMC6397700

[60] Kelly CJ , Makropoulos A , Cordero‐Grande L , Hutter J , Price A , Hughes E , Murgasova M , Teixeira RPAG , Steinweg JK , Kulkarni S , et al. Impaired development of the cerebral cortex in infants with congenital heart disease is correlated to reduced cerebral oxygen delivery. Sci Rep. 2017;7:15088. doi: 10.1038/s41598-017-14939-z 29118365 PMC5678433

[61] Dovjak GO , Hausmaninger G , Zalewski T , Schmidbauer V , Weber M , Worda C , Seidl‐Mlczoch E , Berger‐Kulemann V , Prayer D , Kasprian GJ , et al. Brainstem and cerebellar volumes at magnetic resonance imaging are smaller in fetuses with congenital heart disease. Am J Obstet Gynecol. 2022;227:282.e1–282.e15. doi: 10.1016/j.ajog.2022.03.030 35305961

[62] Schellen C , Ernst S , Gruber GM , Mlczoch E , Weber M , Brugger PC , Ulm B , Langs G , Salzer‐Muhar U , Prayer D , et al. Fetal MRI detects early alterations of brain development in tetralogy of Fallot. Am J Obstet Gynecol. 2015;213:392.e1–392.e7. doi: 10.1016/j.ajog.2015.05.046 26008177

[63] Mlczoch E , Brugger P , Ulm B , Novak A , Frantal S , Prayer D , Salzer‐Muhar U . Structural congenital brain disease in congenital heart disease: results from a fetal MRI program. Eur J Paediatr Neurol. 2013;17:153–160. doi: 10.1016/j.ejpn.2012.07.004 22944287

[64] Ng IHX , Bonthrone AF , Kelly CJ , Cordero‐Grande L , Hughes EJ , Price AN , Hutter J , Victor S , Schuh A , Rueckert D , et al. Investigating altered brain development in infants with congenital heart disease using tensor‐based morphometry. Sci Rep. 2020;10:14909. doi: 10.1038/s41598-020-72009-3 32913193 PMC7483731

[65] Unolt M , Versacci P , Anaclerio S , Lambiase C , Calcagni G , Trezzi M , Carotti A , Crowley TB , Zackai EH , Goldmuntz E , et al. Congenital heart diseases and cardiovascular abnormalities in 22q11.2 deletion syndrome: from well‐established knowledge to new frontiers. Am J Med Genet A. 2018;176:2087–2098. doi: 10.1002/ajmg.a.38662 29663641 PMC6497171

[66] A. Richards A , Garg V . Genetics of congenital heart disease. Curr Cardiol Rev. 2010;6:91–97. doi: 10.2174/157340310791162703 21532774 PMC2892081

[67] Homsy J , Zaidi S , Shen Y , Ware JS , Samocha KE , Karczewski KJ , DePalma SR , McKean D , Wakimoto H , Gorham J , et al. De novo mutations in congenital heart disease with neurodevelopmental and other congenital anomalies. Science. 2015;350:1262–1266. doi: 10.1126/science.aac9396 26785492 PMC4890146

[68] Ji W , Ferdman D , Copel J , Scheinost D , Shabanova V , Brueckner M , Khokha MK , Ment LR . De novo damaging variants associated with congenital heart diseases contribute to the connectome. Sci Rep. 2020;10:7046. doi: 10.1038/s41598-020-63928-2 32341405 PMC7184603

[69] Steinweg JK , Hui GTY , Pietsch M , Ho A , Van Poppel MPM , Lloyd D , Colford K , Simpson JM , Razavi R , Pushparajah K , et al. T2* placental MRI in pregnancies complicated with fetal congenital heart disease. Placenta. 2021;108:23–31. doi: 10.1016/j.placenta.2021.02.015 33798991 PMC7611398

[70] Rychik J , Goff D , McKay E , Mott A , Tian Z , Licht DJ , Gaynor JW . Characterization of the placenta in the newborn with congenital heart disease: distinctions based on type of cardiac malformation. Pediatr Cardiol. 2018;39:1165–1171. doi: 10.1007/s00246-018-1876-x 29728721 PMC6096845

[71] Jones HN , Olbrych SK , Smith KL , Cnota JF , Habli M , Ramos‐Gonzales O , Owens KJ , Hinton AC , Polzin WJ , Muglia LJ , et al. Hypoplastic left heart syndrome is associated with structural and vascular placental abnormalities and leptin dysregulation. Placenta. 2015;36:1078–1086. doi: 10.1016/j.placenta.2015.08.003 26278057 PMC4609616

[72] Matthiesen NB , Henriksen TB , Agergaard P , Gaynor JW , Bach CC , Hjortdal VE , Østergaard JR . Congenital heart defects and indices of placental and fetal growth in a Nationwide study of 924 422 Liveborn infants. Circulation. 2016;134:1546–1556. doi: 10.1161/CIRCULATIONAHA.116.021793 27742737

[73] Salafia CM , Zhang J , Charles AK , Bresnahan M , Shrout P , Sun W , Maas EM . Placental characteristics and birthweight. Paediatr Perinat Epidemiol. 2008;22:229–239. doi: 10.1111/j.1365-3016.2008.00935.x 18426518

[74] Glauser TA , Rorke LB , Weinberg PM , Clancy RR . Congenital brain anomalies associated with the hypoplastic left heart syndrome. Pediatrics. 1990;85:984–990. doi: 10.1542/peds.85.6.984 2339047

[75] Masoller N , Sanz‐Cortés M , Crispi F , Gómez O , Bennasar M , Egaña‐Ugrinovic G , Bargalló N , Martínez JM , Gratacós E . Mid‐gestation brain Doppler and head biometry in fetuses with congenital heart disease predict abnormal brain development at birth. Ultrasound Obstet Gynecol. 2016;47:65–73. doi: 10.1002/uog.14919 26053596

[76] Peng Q , Zhou Q , Zang M , Zhou J , Xu R , Wang T , Zeng S . Reduced fetal brain fissures depth in fetuses with congenital heart diseases: reduced fissures in CHD. Prenat Diagn. 2016;36:1047–1053. doi: 10.1002/pd.4931 27681656

[77] Goldstein IS , Erickson DJ , Sleeper LA , Haynes RL , Kinney HC . The lateral temporal lobe in early human life. J Neuropathol Exp Neurol. 2017;76:424–438. doi: 10.1093/jnen/nlx026 28498956 PMC6251646

[78] Mahle WT , Clancy RR , Moss EM , Gerdes M , Jobes DR , Wernovsky G . Neurodevelopmental outcome and lifestyle assessment in school‐aged and adolescent children with hypoplastic left heart syndrome. Pediatrics. 2000;105:1082–1089. doi: 10.1542/peds.105.5.1082 10790466

[79] Licht DJ , Shera DM , Clancy RR , Wernovsky G , Montenegro LM , Nicolson SC , Zimmerman RA , Spray TL , Gaynor JW , Vossough A . Brain maturation is delayed in infants with complex congenital heart defects. J Thorac Cardiovasc Surg. 2009;137:529–537. doi: 10.1016/j.jtcvs.2008.10.025 19258059 PMC2701902

[80] Tatum WO , Coker SB , Ghobrial M , Abd‐Allah S . The open opercular sign: diagnosis and significance. Ann Neurol. 1989;25:196–199. doi: 10.1002/ana.410250216 2919869

[81] Chen CY , Zimmerman RA , Faro S , Parrish B , Wang Z , Bilaniuk LT , Chou TY . MR of the cerebral operculum: abnormal opercular formation in infants and children. AJNR Am J Neuroradiol. 1996;17:1303–1311. 8871716 PMC8338534

[82] Jørgensen DES , Tabor A , Rode L , Dyre L , Ekelund CK , Hellmuth SG , Macgowan CK , Nørgaard LN , Seed M , Sundberg K , et al. Longitudinal brain and body growth in fetuses with and without transposition of the great arteries: quantitative volumetric magnetic resonance imaging study. Circulation. 2018;138:1368–1370. doi: 10.1161/CIRCULATIONAHA.118.034467 30354424

[83] Xuegang L , Chao S , Kangwu W , Yiyao L , Guixin D , Xiaohong L , Wei S , Junxiang Z . Porcine model of congenital heart defect with decreased pulmonary blood flow. Cell Biochem Biophys. 2011;61:725–730. doi: 10.1007/s12013-011-9246-9 21805397

[84] Ishibashi N , Scafidi J , Murata A , Korotcova L , Zurakowski D , Gallo V , Jonas RA . White matter protection in congenital heart surgery. Circulation. 2012;125:859–871. doi: 10.1161/CIRCULATIONAHA.111.048215 22247493 PMC3288390

[85] Morton PD , Korotcova L , Lewis BK , Bhuvanendran S , Ramachandra SD , Zurakowski D , Zhang J , Mori S , Frank JA , Jonas RA , et al. Abnormal neurogenesis and cortical growth in congenital heart disease. Sci Transl Med. 2017;9:eaah7029. doi: 10.1126/scitranslmed.aah7029 28123074 PMC5467873

[86] Kostovic I , Judas M . Prolonged coexistence of transient and permanent circuitry elements in the developing cerebral cortex of fetuses and preterm infants. Dev Med Child Neurol. 2006;48:388–393. doi: 10.1017/S0012162206000831 16608549

[87] Kostović I , Jovanov‐Milosević N . The development of cerebral connections during the first 20–45 weeks' gestation. Semin Fetal Neonatal Med. 2006;11:415–422. doi: 10.1016/j.siny.2006.07.001 16962836

[88] Wilson S , Pietsch M , Cordero‐Grande L , Price AN , Hutter J , Xiao J , McCabe L , Rutherford MA , Hughes EJ , Counsell SJ , et al. Development of human white matter pathways in utero over the second and third trimester. Proc Natl Acad Sci USA. 2021;118:e2023598118. doi: 10.1073/pnas.2023598118 33972435 PMC8157930

[89] Clouchoux C , Guizard N , Evans AC , Du Plessis AJ , Limperopoulos C . Normative fetal brain growth by quantitative in vivo magnetic resonance imaging. Am J Obstet Gynecol. 2012;206:173.e1–173.e8. doi: 10.1016/j.ajog.2011.10.002 PMC13054723 22055336

[90] Ortinau CM , Mangin‐Heimos K , Moen J , Alexopoulos D , Inder TE , Gholipour A , Shimony JS , Eghtesady P , Schlaggar BL , Smyser CD . Prenatal to postnatal trajectory of brain growth in complex congenital heart disease. NeuroImage Clin. 2018;20:913–922. doi: 10.1016/j.nicl.2018.09.029 30308377 PMC6178192

[91] Khan S , Rollins CK , Ortinau CM , Afacan O , Warfield SK , Gholipour A . Tract‐specific group analysis in fetal cohorts using in utero diffusion tensor imaging. Med Image Comput Comput‐Assist Interv. 2018;11072:28–35. 32869014 10.1007/978-3-030-00931-1_4 PMC7456441

[92] Ren J‐Y , Ji H , Zhu M , Dong S‐Z . DWI in brains of fetuses with congenital heart disease: a case‐control MR imaging study. Am J Neuroradiol. 2021;42:2040–2045. doi: 10.3174/ajnr.A7267 34475195 PMC8583281

[93] Song J‐G , Sun C , Zhu M , Zhu J‐X , Zhang N , Wang G‐B , Zhao B . Regional changes in brain apparent diffusion coefficient in fetuses with complex congenital heart disease and normal pregnancy assessed using diffusion‐weighted imaging. Front Neurol. 2023;14:1136633. doi: 10.3389/fneur.2023.1136633 37351264 PMC10283352

[94] Wilson S , Pietsch M , Cordero‐Grande L , Christiaens D , Uus A , Karolis VR , Kyriakopoulou V , Colford K , Price AN , Hutter J , et al. Spatiotemporal tissue maturation of thalamocortical pathways in the human fetal brain. eLife. 2023;12:e83727. doi: 10.7554/eLife.83727 37010273 PMC10125021

[95] Claessens NHP , Algra SO , Ouwehand TL , Jansen NJG , Schappin R , Haas F , Eijsermans MJC , De Vries LS , Benders MJNL ; CHD Lifespan Study Group Utrecht . Perioperative neonatal brain injury is associated with worse school‐age neurodevelopment in children with critical congenital heart disease. Dev Med Child Neurol. 2018;60:1052–1058. doi: 10.1111/dmcn.13747 29572821

[96] Porayette P , Madathil S , Sun L , Jaeggi E , Grosse‐Wortmann L , Yoo S‐J , Hickey E , Miller SP , Macgowan CK , Seed M . MRI reveals hemodynamic changes with acute maternal hyperoxygenation in human fetuses with and without congenital heart disease. Prenat Diagn. 2016;36:274–281. doi: 10.1002/pd.4762 26701792

[97] Stegeman R , Nijman M , Breur JMPJ , Groenendaal F , Haas F , Derks JB , Nijman J , van Beynum IM , Taverne YJHJ , Bogers AJJC , et al. CeRebrUm and CardIac protection with ALlopurinol in neonates with critical congenital heart disease requiring cardiac surgery with cardiopulmonary bypass (CRUCIAL): study protocol of a phase III, randomized, quadruple‐blinded, placebo‐controlled. Dutch Multicenter Trial Trials. 2022;23:174. doi: 10.1186/s13063-022-06098-y 35197082 PMC8867620

[98] Gaynor JW , Moldenhauer JS , Zullo EE , Burnham NB , Gerdes M , Bernbaum JC , D'Agostino JA , Linn RL , Klepczynski B , Randazzo I , et al. Progesterone for neurodevelopment in fetuses with congenital heart defects. JAMA Netw Open. 2024;7:e2412291. doi: 10.1001/jamanetworkopen.2024.12291 38805228 PMC11134212

