# Supplementary material for: Structural Covariance Networks in the Fetal Brain Reveal Altered Neurodevelopment for Specific Subtypes of Congenital Heart Disease
Source: J Am Heart Assoc. 2024 Oct 25;13(21):e035880. doi: 10.1161/JAHA.124.035880 (PMC11935691; doi:10.1161/JAHA.124.035880)
Supplement: Supplementary file 1 — Tables S1–S2 Figure S1 [file JAH3-13-e035880-s001.pdf]

SUPPLEMENTAL MATERIAL

Table S1. A summary of all diagnoses included in this study, along with the rationale for grouping Congenital Heart Disease (CHD) diagnoses into cerebral substate delivery categories[14]

Diagnosis	Potential (cerebrovascular) physiology associated with diagnosis	Factors considered when assigning to group	Rationale (Group)
Normal (Control)	-	-	Normal cerebral substrate delivery (0)
Right aortic arch	Normal streaming, forward flow through left heart/aortic isthmus	Underlying anatomy	No anticipated effects of CHD on cerebral substrate delivery (1)
Double aortic arch	Normal streaming, forward flow through left heart/aortic isthmus	Underlying anatomy	No anticipated effects of CHD on cerebral substrate delivery (1)
Coarctation of the aorta, (CoA (+))	Normal streaming, forward flow through left heart/aortic isthmus<br><br>Normal streaming, forward flow through left heart, reversal of flow at isthmus	Underlying anatomy; phase contrast flow; echocardiography	No anticipated effects of CHD on cerebral substrate delivery (1)<br><br>Mild reduction in cerebral substrate delivery (2)
Antenatally suspected coarctation of the aorta, not requiring surgery or intervention in the neonatal period, CoA (-)	Normal streaming, forward flow through left heart/aortic isthmus<br><br>Normal streaming, forward flow through left heart, reversal of flow at isthmus	Underlying anatomy; phase contrast flow; echocardiography	No anticipated effects of CHD on cerebral substrate delivery (1)<br><br>Mild reduction in cerebral substrate delivery (2)
Interrupted aortic arch	Normal streaming, forward flow through left heart to carotid arteries	Underlying anatomy	No anticipated effects of CHD on cerebral substrate delivery
Partial anomalous pulmonary venous drainage	Normal streaming, forward flow through left heart/aortic isthmus	Underlying anatomy	No anticipated effects of CHD on cerebral substrate delivery (1)

Ventricular septal defect	Normal streaming, forward flow through left heart/aortic isthmus	Underlying anatomy	No anticipated effects of CHD on cerebral substrate delivery (1)
Anomalous left subclavian artery	Normal streaming, forward flow through left heart/aortic isthmus	Underlying anatomy	No anticipated effects of CHD on cerebral substrate delivery (1)
Atrioventricular septal defect + coarctation of the aorta	Normal streaming, forward flow through left heart/aortic isthmus	Underlying anatomy	No anticipated effects of CHD on cerebral substrate delivery (1)
Dilated aorta	Normal streaming, forward flow through left heart/aortic isthmus	Underlying anatomy	No anticipated effects of CHD on cerebral substrate delivery (1)
Ventricular asymmetry	Normal streaming, forward flow through left heart/aortic isthmus	Underlying anatomy	No anticipated effects of CHD on cerebral substrate delivery (1)
Left atrial diverticulum	Normal streaming, forward flow through left heart/aortic isthmus	Underlying anatomy	No anticipated effects of CHD on cerebral substrate delivery (1)
Left atrial aneurysm	Normal streaming, forward flow through left heart/aortic isthmus	Underlying anatomy	No anticipated effects of CHD on cerebral substrate delivery (1)
Persistent left superior vena cava	Normal streaming, forward flow through left heart/aortic isthmus	Underlying anatomy	No anticipated effects of CHD on cerebral substrate delivery (1)
Cardiac apex to the right	Normal streaming, forward flow through left heart/aortic isthmus	Underlying anatomy	No anticipated effects of CHD on cerebral substrate delivery (1)
Mesocardia	Normal streaming, forward flow through left heart/aortic isthmus	Underlying anatomy	No anticipated effects of CHD on cerebral substrate delivery (1)
Cardiomegaly	Normal streaming, forward flow through left heart/aortic isthmus	Underlying anatomy	No anticipated effects of CHD on cerebral substrate delivery (1)
Cardiac mass	Normal streaming, forward flow through left heart/aortic isthmus	Underlying anatomy	No anticipated effects of CHD on cerebral substrate delivery (1)
Bilateral superior vena cava	Normal streaming, forward flow	Underlying anatomy	No anticipated effects of CHD on

	through left heart/aortic isthmus		cerebral substrate delivery (1)
Total anomalous pulmonary venous drainage	Minimal if any effect on placental streaming, forward flow through left heart to carotid arteries	Underlying anatomy; phase contrast flow; echocardiography	No anticipated effects of CHD on cerebral substrate delivery (1)
Pulmonary Stenosis	Normal streaming, forward flow through left heart to carotid arteries (mild stenosis)<br><br>Reduced flow through the right heart with likely increased right to left atrial or ventricular shunting (moderate + stenosis + presence of VSD relevant).	Underlying anatomy; echocardiography	No anticipated effects of CHD on cerebral substrate delivery (1)<br><br>Mild reduction in cerebral substrate delivery (2)
Congenitally corrected TGA (ccTGA)	Normal streaming, forward flow through left heart to carotid arteries<br><br>Abnormal cardiac streaming, forward flow through left heart to carotid arteries<br><br>Normal streaming, forward flow through left heart, reversal of flow at isthmus	Underlying anatomy; echocardiography	No anticipated effects of CHD on cerebral substrate delivery (1)<br><br>Mild reduction in cerebral substrate delivery (2)<br><br>Mild reduction in cerebral substrate delivery (2)
Tetralogy of Fallot + absent pulmonary valve	Likely increased right to left atrial shunting	Underlying anatomy; phase contrast flow; echocardiography	Mild reduction in cerebral substrate delivery (2)
Aortic stenosis	Normal streaming, forward flow through left heart to carotid arteries<br>Reduced aortic flow with reversal at isthmus	Underlying anatomy; echocardiography	No anticipated effects of CHD on cerebral substrate delivery (1)<br><br>Mild reduction in cerebral substrate

			delivery (2)
Coarctation of the aorta with aortic stenosis	Normal streaming, forward flow through left heart to carotid arteries Reduced aortic flow with reversal at isthmus	Underlying anatomy; phase contrast flow; echocardiography	Mild reduction in cerebral substrate delivery (2)
Unbalanced AVSD	Normal streaming, forward flow through left heart to carotid arteries Reduced aortic flow with reversal at isthmus	Underlying anatomy; phase contrast flow; echocardiography	Mild reduction in cerebral substrate delivery (2)
Tetralogy of Fallot (including double outlet right ventricle)	Reduced flow through right heart with increased right to left atrial or ventricular shunting	Underlying anatomy	Mild reduction in cerebral substrate delivery (2)
Tricuspid dysplasia	Reduced flow through right heart with increased right to left atrial shunting	Underlying anatomy	Mild reduction in cerebral substrate delivery (2)
Common arterial trunk	Complete admixture of placental and fetal systemic venous blood	Underlying anatomy	Moderately reduced SaO2 in carotid arteries (3)
Hypoplastic left heart syndrome	Complete admixture of placental and fetal systemic venous blood	Underlying anatomy	Moderately reduced SaO2 in carotid arteries (3)
Pulmonary Atresia	Complete admixture of placental and fetal systemic venous blood	Underlying anatomy	Moderately reduced SaO2 in carotid arteries (3)
Hypoplastic right heart syndrome	Complete admixture of placental and fetal systemic venous blood	Underlying anatomy	Moderately reduced SaO2 in carotid arteries (3)
Double outlet right ventricle + mitral atresia	Complete admixture of placental and fetal systemic venous blood	Underlying anatomy	Moderately reduced SaO2 in carotid arteries (3)
Double outlet right ventricle +	Complete admixture of placental	Underlying anatomy	Moderately reduced SaO2 in carotid

pulmonary atresia	and fetal systemic venous blood		arteries (3)
Critical aortic stenosis with intact atrial septum.	Complete admixture of placental and fetal systemic venous blood	Underlying anatomy	Moderately reduced SaO2 in carotid arteries (3)
Transposition of the great arteries + double outlet right ventricle + coarctation of the aorta	Reversal of normal fetal streaming with forward flow at the aortic isthmus	Underlying anatomy	Severely reduced SaO2 in carotid arteries (4)
Transposition of the great arteries	Reversal of normal fetal streaming	Underlying anatomy	Severely reduced SaO2 in carotid arteries (4)

Table S2. Number of subjects with each Congenital Heart Disease (CHD) Diagnosis.

CHD Diagnosis	Abbreviation	n
Absent Pulmonary Valve	APV	4
Anomalous Left Subclavian Artery	ALSA	1
Aortic Stenosis	AS	3
Atrial Septal Aneurysm	ASA	1
Atrioventricular Septal Defect	AVSD	1
Atrioventricular Septal Defect + Coarctation of the Aorta (+)	AVSD + CoA (+)	3
Cardiac Apex to the Right	-	1
Cardiac Mass	-	3
Cardiomegaly	-	2
Congenitally Corrected Transposition of the Great Arteries	ccTGA	2
Coarctation of the Aorta (-)	CoA (-)	47
Coarctation of the Aorta (+)	CoA (+)	58
Common Arterial Trunk	CAT	6
Control	-	67
Double Aortic Arch	DAA	27
Dilated Aorta	-	8
Diverticulum	-	2
Double Outlet Right Ventricle	DORV	3
Double Outlet Right Ventricle + Pulmonary Atresia	DORV + PA	1
Double Outlet Right Ventricle + Transposition of the Great Arteries	DORV + TGA	1
Hypoplastic Left Heart Syndrome	HLHS	25
Hypoplastic Right Heart Syndrome	HRHS	1
Interrupted Aortic Arch	IAA	3
Left Atrial Aneurysm	-	5
Mesocardia	-	2
Mitral Atresia + Double Outlet Right Ventricle	MA + DORV	4
Normal	-	1
Partial Anomalous Pulmonary Venous Drainage	PAPVD	2
Persistent Left Superior Vena Cava	PLSVC	6

Pulmonary Atresia	PA	8
Pulmonary Stenosis	PS	2
Right Aortic Arch	RAA	88
Situs Solitus	-	1
Total Anomalous Pulmonary Venous Drainage	TAPVD	1
Transposition of the Great Arteries	TGA	22
Tetralogy of Fallot	ToF	13
Tetralogy of Fallot + Absent Pulmonary Valve	ToF + APV	1
Tricuspid Dysplasia	-	1
Unbalanced Atrioventricular Septal Defect, Interrupted Aortic Arch	Unbalanced AVSD, IAA	1
Ventricular Asymmetry	-	1
Ventricular Septal Defect	VSD	2

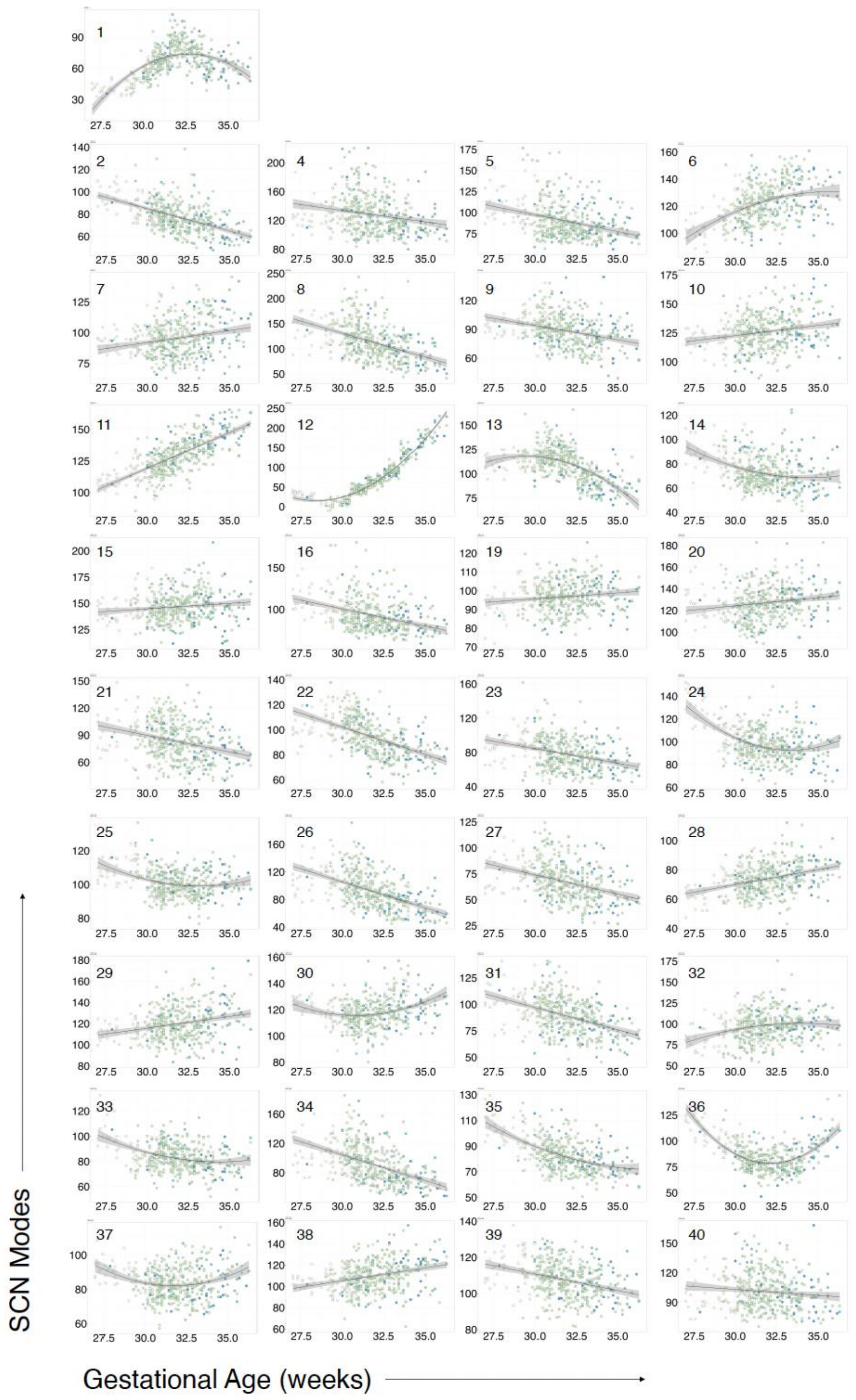

Figure S1. For 37 Structural Covariance Networks (SCNs) Gestational Age (GA) was a significant predictor of variation between individuals, best fit was determined using AIC, either a linear or 2nd order polynomial relationship.
